# The Influence of Pentraxin 3 on the Ovarian Function and Its Impact on Fertility

**DOI:** 10.3389/fimmu.2018.02808

**Published:** 2018-11-29

**Authors:** Antonella Camaioni, Francesca Gioia Klinger, Luisa Campagnolo, Antonietta Salustri

**Affiliations:** Histology and Embryology Section, Department of Biomedicine and Prevention, University of Rome “Tor Vergata,” Rome, Italy

**Keywords:** PTX3, fertility, PCOS, ovarian disorders, theca cells, follicle growth, cumulus matrix

## Abstract

Follicular development is a highly coordinated process that in humans takes more than 6 months. Pituitary gonadotropins and a variety of locally produced growth factors and cytokines are involved in determining a precise sequence of changes in cell metabolism, proliferation, vascularization, and matrix remodeling in order to obtain a follicle with full ovulatory and steroidogenic capability. A low-grade inflammation can alter such processes leading to premature arrest of follicular growth and female reproductive failure. On the other hand, factors that are involved in inflammatory response as well as in innate immunity are physiologically upregulated in the follicle at the final stage of maturation and play an essential role in ovulation and fertilization. The generation of pentraxin 3 (PTX3) deficient mice provided the first evidence that this humoral pattern recognition molecule of the innate immunity has a non-redundant role in female fertility. The expression, localization, and molecular interactions of PTX3 in the periovulatory follicle have been extensively studied in the last 10 years. In this review, we summarize findings demonstrating that PTX3 is synthesized before ovulation by cells surrounding the oocyte and actively participates in the organization of the hyaluronan-rich provisional matrix required for successful fertilization. Data in humans tend to confirm these findings, indicating PTX3 as a biomarker of oocyte quality. Moreover, we discuss the emerging evidence that in humans altered PTX3 systemic levels, determined by genetic variations and/or low-grade chronic inflammation, can also impact the growth and development of the follicle and affect the incidence of ovarian disorders.

## Introduction

The ovary is the organ assigned to the cyclic production of a mature egg as well as of steroid hormones that, acting locally and systemically, influence female fertility and metabolic activity of many tissues. These functions are accomplished by ovarian follicles in which growth and maturation of the oocyte occur in parallel with proliferation and differentiation of epithelial somatic cells, named granulosa cells. During its development, the follicle induces the formation of a specialized connective tissue, the theca layer, which organizes an extended network of blood vessels supplying the avascular multilayered follicle cells with nutrients, oxygen, and pituitary gonadotropins. Theca cells are also directly involved in the ovarian endocrine function producing androgens that granulosa cells convert to estrogens. As expected in a connective tissue, immune cells are present in the theca layer of the follicle and their number and type change at different follicle stages. Strong evidences indicate that the immune system plays an important role in the physiology of the ovary (1). Depletion of macrophages/dendritic cells in CD11c-diptheria toxin receptor transgenic mice resulted in loss of ovarian vascular integrity, reduction in mature follicles and impaired ovulation (2–4). Immune cells are also essential for vasculature invasion of luteinized theca and granulosa cells and for the formation of the corpus luteum (2, 3, 5, 6). Of note, before ovulation also granulosa cells acquire an inflammatory and immune–like phenotype producing prostaglandins, inflammatory cytokines, chemokines, and innate immune components that play an essential role in ovulation and fertilization (7–9). Uncontrolled systemic pro-inflammatory conditions alter ovarian homeostasis and have a negative impact on follicular dynamics. Indeed, it has been proposed that even a low-grade chronic inflammation and a small imbalance between pro- and anti-inflammatory cytokines play a role in the pathogenesis of polycystic ovarian syndrome (PCOS) (10, 11), characterized by follicle growth alteration and oligo or anovulatory cycles.

Here we will review the ovarian expression and biological function of pentraxin 3 (PTX3), a multifunctional protein implicated in innate immunity response, regulation of inflammation, angiogenesis and formation and remodeling of the extracellular matrix.

## The long pentraxin PTX3: gene, structure and ligands

**Figure 1:**
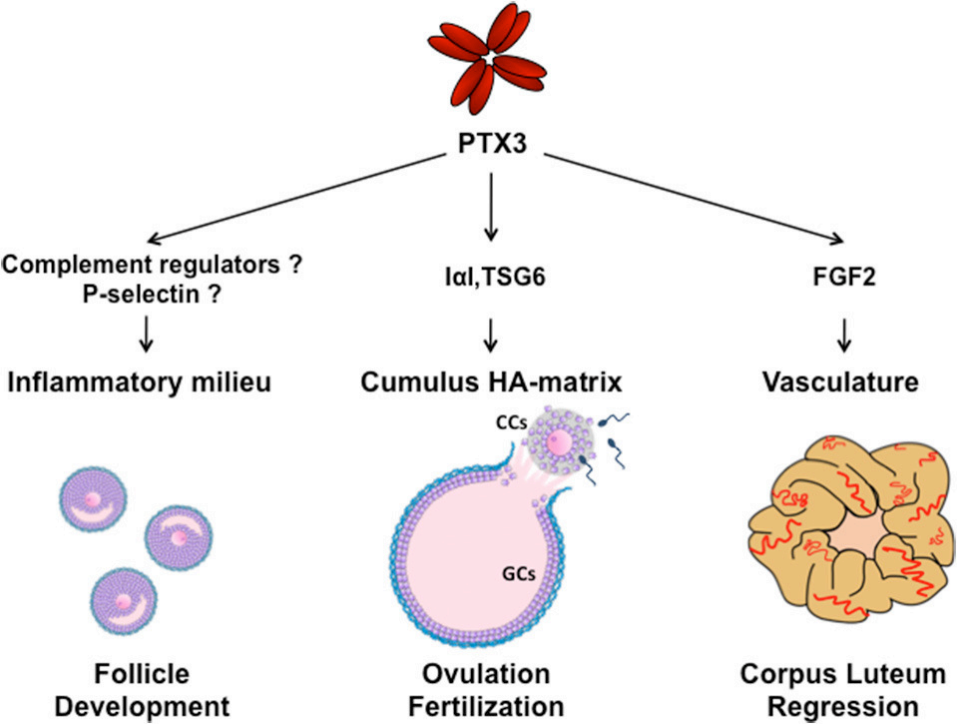
Schematic representation of molecular interactions and functions of PTX3 during folliculogenesis. SNP haplotypes of PTX3 and altered circulating PTX3 levels in reproductive disorders suggest a role of PTX3 in the control of ovarian immune milieu during follicle development. In the preovulatory follicle, PTX3 is expressed by CCs and interacts with IαI and TSG6 for organizing the HA matrix, which is essential for oocyte ovulation and fertilization. After ovulation, PTX3 is expressed by stromal and endothelial cells of the corpus luteum and, by sequestering FGF2, is involved in vasculature involution during its regression. (CCs, cumulus cells; GCs, granulosa cells).

PTX3 is a glycoprotein assembled to form an octameric complex stabilized by intermolecular disulfide bonds (12–14). The primary sequence of PTX3 is highly conserved among species and consists of two structural domains: a C-terminal region showing homology with the classical short pentraxin C-reactive protein (CRP) and serum amyloid P component (SAP), and a unique N-terminal domain that has no homology with any other known protein (15). The PTX3 gene is arranged in three exons, with the first and second coding the signal peptide and the N-terminal domain, and the third exon coding the C-terminal pentraxin domain. PTX3 is released by peripheral blood leukocytes and myeloid dendritic cells following stimulation with pro-inflammatory cytokines (IL-1 and TNF-α), agonists of TLR or microbial components (16). PTX3 production is also stimulated in myeloid cells by the anti-inflammatory cytokine IL-10, which is essential for damping inflammation and preventing tissue damage (17). Human neutrophils store PTX3 in lactoferrin-positive granules and rapidly release it at the inflammatory site (18). Other cell types produce PTX3 locally in response to inflammatory conditions and appropriate stimuli: smooth muscle cells, fibroblasts, adipocytes, chondrocytes, mesangial, endothelial, mesenchymal stroma cells, and ovarian cells (19). PTX3 has multifunctional properties for its capacity to interact with different types of ligands (19). In particular, PTX3 plays a non-redundant role in innate immunity by opsonizing selected pathogens and binding and facilitating clearance of apoptotic cells (20, 21). PTX3 modulates the inflammatory reaction by binding elements of the complement cascade and regulating complement activation. It interacts with surface-bound C1q, ficolin 1, ficolin 2 and mannose binding lectin and activates the classical and lectin complement pathways (22–26). On the other hand, PTX3 modulates the alternative complement pathway by recruiting the factor H and enhancing the inactivation of C3b to iC3b, both recognized by the leukocyte receptor CD11/CD18 (27, 28). Coating of microbes and apoptotic cells by PTX3 would help phagocytosis during infection and sterile inflammation without inducing excessive complement activation and tissue harm. PTX3 also binds Fibroblast Growth Factor-2 (FGF2) and sequesters the growth factor in an inactive form, thus modulating angiogenesis in various physio-pathological conditions (29). Interaction of PTX3 with plasminogen and fibrin in wounding or injured tissue matrix has been recently demonstrated. It allows migration of macrophages and mesenchymal stroma cells by promoting pericellular fibrinolysis (30). PTX3 is expressed at specific sites and times during the ovarian cycle and play different roles (Figure 1).

## PTX3 expression in the ovary

PTX3 is specifically expressed by a small group of granulosa cells surrounding the oocyte, namely cumulus cells, following LH or hCG stimulation of preovulatory follicles. This cell subpopulation differs in many aspects from the majority of granulosa cells and have different fate. Granulosa cells produce PGE2 and a series of EGF-like growth factors and cytokines under the LH/hCG stimulation. They upregulate proteolytic activity in the theca, leading to matrix degradation, cell death and break at the site of follicle wall facing the ovarian surface (9). Conversely, the oocyte modulates the response of cumulus cells to granulosa cell-derived factors mentioned above thereby inducing these cells to synthesize an extensive extracellular matrix, which facilitates the oocyte release and *in vivo* fertilization (31–33) (Figure 1). Mechanical analysis of the rheological properties of this matrix by colloidal-probe atomic force microscopy showed that it is extremely soft and with mucoelastic characteristics (34). The main component of such peculiar matrix is hyaluronan (HA), a long polysaccharide synthesized by HAS2 and organized by proteins in a highly hydrated mesh-like structure, which increases the space among the cells and consequently the overall volume of the cumulus cell oocyte complex (COC) (35, 36). For this characteristic, the process is named cumulus expansion.

PTX3 is one of the most upregulated genes by the oocyte in the mouse cumulus cells before ovulation and is involved in cumulus matrix formation (37). Deletion of *Ptx3* gene in mice results in female infertility for the failure of oocyte fertilization due to ovulation of abnormal COCs. In COC ovulated by *Ptx3* deficient mice cumulus cells appear to form a uniform unstable mass, rather than surrounding a central positioned oocyte, and quickly disperse in the oviduct (38). *In vitro* studies demonstrated that *Ptx3*^−/−^ COC induced to expand *in vitro* is able to synthesize HA at the normal rate but this polymer is released into the medium, instead being organized in a matrix. The normal matrix phenotype can be restored *in vitro* by stimulating *Ptx3*^−/−^ COCs in the presence of the recombinant full length PTX3 (rhPTX3) or the recombinant N-terminal region (rhNter-PTX3), but not by the C-terminal fragment of the protein (39). Therefore, although short pentraxins have the ability to bind to some matrix components (40), the action of PTX3 is distinct being fully exerted through the unique sequence of the molecule, then assigning a specific role to PTX3 in HA matrix organization (Figure 1).

**Figure 2:**
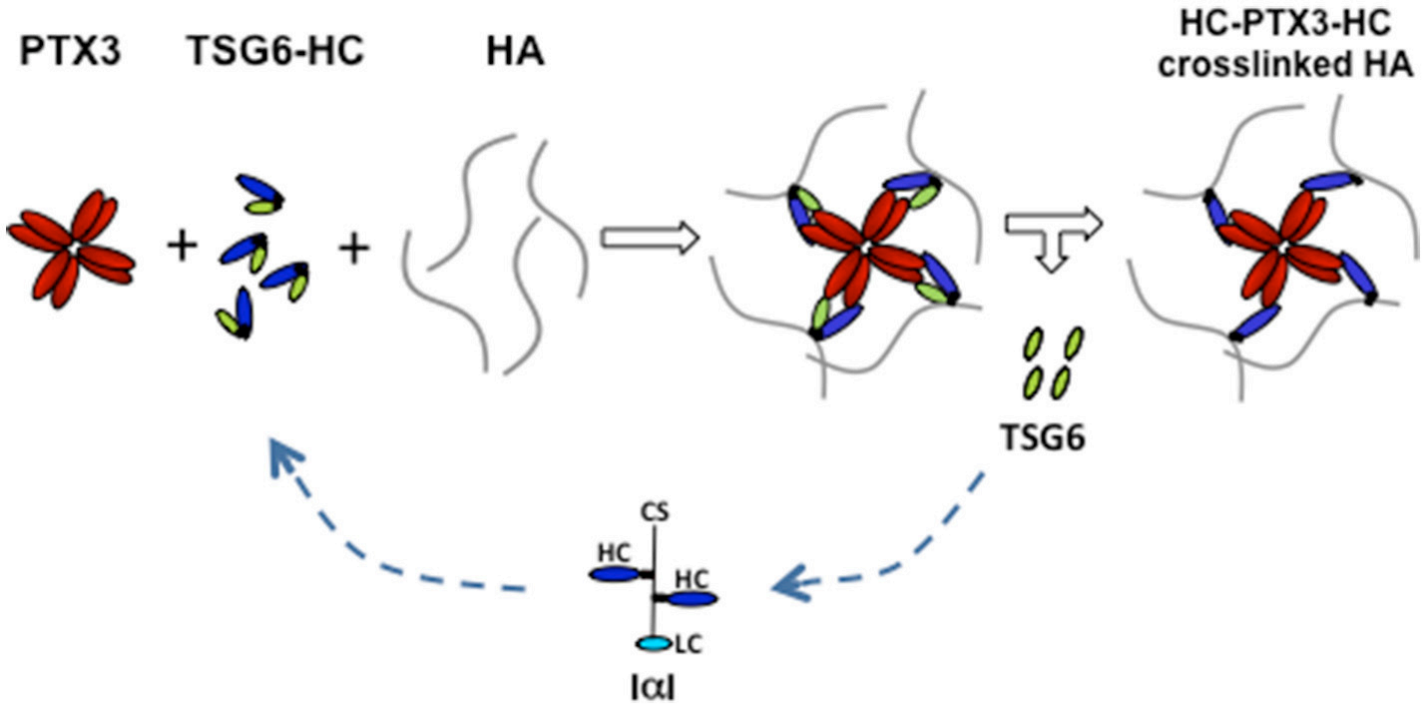
Proposed model of PTX3/HC/TSG6/HA interactions in cumulus matrix. During cumulus matrix formation, the binding of HA-linked HCs to the multimeric PTX3 molecule allows the crosslinking of several HA strands. TSG6, that catalyzes the transfer of HCs from CS of IαI to HA through the formation of an intermediate complex (TSG6-HC), also binds to PTX3. These interactions facilitate the simultaneous and coordinated integration of HCs and PTX3 within the cumulus matrix. (CS, chondroitin sulfate; LC, light chain; HC, heavy chain; HA, hyaluronan).

PTX3 does not bind to HA but can bind to inter-α-trypsin inhibitor (IαI) proteoglycan and tumor necrosis factor-inducible gene 6 (TSG-6) protein (38, 39). The former is mainly synthesized by the liver and circulating in the blood (41), while the latter is synthesized by granulosa cells and cumulus cells concomitantly to HA and PTX3 (38). IαI is a peculiar proteoglycan consisting in a protein carrying one chondroitin sulfate (CS) chain, called bikunin, to which two homologous proteins, named heavy chains (HCs), are linked to the CS in the Golgi through an ester bond (41). The increased vessel permeability in the periovulatory follicle facilitates the diffusion of IαI in the follicular fluid (42, 43) and the HCs are translocated from the CS to the elongating HA polymers by TSG-6, which catalyzes the transfer via a transesterification reaction. Blocking HC integration in the cumulus matrix by the deletion of *bikunin* (which prevents the assembly of intact IαI) or *Tsg6* gene in mice produces female sterility and cumulus matrix instability, as in *Ptx3* null mice (38, 44, 45). PTX3 does not influence the transfer of HCs to HA, but it interacts with HCs in biological context as assessed by co-localization and co-precipitation from COC matrix extracts (39). In addition, the HC binding site resides in the PTX3 N-terminal domain and a monoclonal antibody inhibiting their interaction neutralizes full-length rhPTX3 in restoring normal phenotype in *Ptx3* deficient COCs (39). Site direct mutagenesis of cysteines forming disulphide bonds revealed the relevance of PTX3 multimeric state in matrix formation and suggested that its octameric structure provides at least four binding sites for HCs (12, 46). Thus, it has been hypothesized that multimeric PTX3 might stabilize the HA network by binding several HCs covalently linked to distinct HA molecules, acting as a “node” (39, 46) (Figure 2). TSG-6 has an HA binding capacity and PTX3 has multiple binding sites for this protein, as found for HCs (46, 47). However, several lines of evidence do not support the possibility that TSG-6 directly participates in crosslinking HA. First, matrix formation is not inhibited by HA hexasaccharides competing with the TSG-6 binding to HA (48). In agreement, mutants of TSG-6 with highly reduced HA binding capacity do support matrix assembly of *Tsg-6* deficient COC *in vitro* (49). Finally, during expansion, all TSG-6 molecules form covalent complexes with individual HCs that act as intermediates in the transfer reaction. On these bases, it has been proposed that “the binding of TSG-6 to PTX3 might favor the interaction of PTX3 with HCs committed to link with HA (those in TSG6-HC complexes), leading to the integration of PTX3 into the matrix at the same time as, and in coordinate fashion to, HCs” (39) (Figure 2). This hypothesis found a strong support in an *in vitro* binding assay where PTX3, IαI, and TSG-6 are added to an immobilized HA film in a controlled sequence. The results demonstrated that PTX3 can be incorporated into the HA film only if it is pre-mixed with IαI and TSG-6 (47).

Interestingly, HA-HC-PTX3 complex is also formed in human amniotic membrane and is reported to exert anti-inflammatory and anti-scarring actions in inflamed tissues by inhibiting M1 macrophages infiltration and further polarizing them toward M2 phenotype (50). Then, it is possible that HC-PTX3 interaction in the cumulus matrix, besides having a structural role, could also protect the oocyte by dampening the activity of leukocytes in the reproductive tracts and preserving the microenvironment for optimal fertilization. The presence of PTX3 in the cumulus matrix and in the follicular fluids aspirated from patients undergoing IVF suggest that this molecule might have the same role in human female fertility (38).

Following the expulsion of the COC, theca cells, fibroblasts, and endothelial cells invade the granulosa cell layers by initiating the formation of the corpus luteum. Luteal theca and granulosa cells produce progesterone, which sustains embryo implantation. An intensive formation of blood vessels occurs during the maturation of the corpus luteum producing one of the greatest rates of blood flow of any tissue in the body (51). In the absence of pregnancy, this gland loses its function and structure and undergo to regression. Transcriptome analysis of the bovine corpus luteum isolated from animals treated with prostaglandin F2alpha, the physiological inducer of luteolysis in most domestic animals and likely in primates, showed a significant upregulation of PTX3 expression at the early stage of luteal regression, concomitant with FGF2 expression. Either luteal endothelial and steroidogenic cells showed this ability *in vitro* (52, 53). It has been then suggested that PTX3 might participate in the involution of microvasculature during corpus luteum regression by sequestering FGF2 and preventing its pro-angiogenic activity (53, 54) (Figure 1). In contrast, one study performed in sheep reported that PTX3 expression is downregulated during physiological regression of corpus luteum while it is maintained in gravidic corpus luteum (55). If these conflicting results depend on differences among species or between physiological and experimental-induced luteolysis remains to be determined. In any event, the evidence that PTX3 is expressed and modulated in the corpus luteum urges further studies.

## Immune cell-derived PTX3 and human ovarian disorders

Polycystic ovary syndrome (PCOS) is one of the most common endocrine disorders affecting 5-10% of premenopausal women. It is characterized by hyperandrogenism, oligo- anovulation and polycystic ovary, often associated with obesity and other metabolic dysfunctions (56). The syndrome is caused by the pronounced increase in the number of small-mid antral follicles (2–9 mm) unable to complete the growth (17–20 mm) and proceed to maturation. Theca layer is thicker and the cells produce an excess of androgens. Such alteration in the development of ovarian follicles is associated to low-grade inflammation (10, 11) and local infiltration of immune cells in the theca layer (57–61). Of note, it has been reported that the short CRP and classical pro-inflammatory cytokines levels are slightly but significantly elevated while the long PTX3 level is reduced in the blood of PCOS and overweight women (62–67). Based on the protective and anti-inflammatory role recently assigned to PTX3 (68), it is likely that reduced PTX3 levels would increase the sensitivity of the ovary to the inflammatory status. In agreement, a lower level of circulating IL-10 was found in PCOS patients and linked to higher risk to develop the ovarian hyperstimulation syndrome (69), an exacerbated reaction to hormone stimulation in assisted reproductive programs characterized by local and generalized increased capillary permeability and enhanced production of inflammatory cytokines by immune cells. These findings further support the importance of appropriate balance of immune cell types in controlling and promoting follicle development (Figure 1).

The altered mechanisms underlying the excessive follicle formation in PCOS has not been clearly understood. The overexpression of LH receptor mRNA in granulosa cells and under-expression of GDF9 by the oocyte in PCOS follicles compared to normal follicles of the same size suggest a premature terminal differentiation (70, 71) and closely resemble the conditions promoting polyovulation and dizygotic twinning in sheep (72, 73). Noteworthy, a study on PTX3 single-nucleotide polymorphisms (SNPs) performed in Gambia, where the dizygotic twinning incidence is the highest worldwide (74), showed that a specific three SNP haplotype GAG (at positions rs2305619, rs3816527 and rs1840680) is more frequent in mothers of dizygotic twins compared to women without a history of twinning (75). In addition, in another study performed on Ghanaian women, the same haplotype positively correlates with the number of children given birth during the lifetime (76). Unfortunately, the twinning frequency was not analyzed in this study, but Ghana is another African state with high twinning incidence (77). Moreover, the GAG haplotype confers resistance to *Mycobacterium tuberculosis* and decreased the risk of pulmonary tuberculosis (78), indicating that the protein is functional.

All together, these data indicate that altered expression of PTX3 can influence the ovarian microenvironment and alter folliculogenesis, likely deregulating the fine-tuned inflammatory milieu of the follicle (Figure 1).

## Author contributions

AS prepared the manuscript draft in consultation with AC. FK and LC participated in revising it critically for important intellectual content. All authors contributed to and approved the final version of the manuscript.

### Conflict of interest statement

The authors declare that the research was conducted in the absence of any commercial or financial relationships that could be construed as a potential conflict of interest.
